# Diffusion-weighted MRI improves response assessment after definitive radiotherapy in patients with NSCLC

**DOI:** 10.1186/s40644-021-00384-9

**Published:** 2021-01-21

**Authors:** Jagoda Philippe, Fleckenstein Jochen, Sonnhoff Mathias, Schneider Günther, Ruebe Christian, Buecker Arno, Stroeder Jonas

**Affiliations:** 1 Clinic for Diagnostic and Interventional Radiology, Saarland University Medical Center, Kirrberger Str. 1, 66421 Homburg, Saar Germany; 2 Department of Radiotherapy and Radiation Oncology, Saarland University Medical Center, Kirrberger Str. Geb. 6.5, 66421 Homburg, Saar Germany

**Keywords:** Tomography, Spiral computed, Magnetic resonance imaging, Functional magnetic resonance imaging, Radiotherapy, Image-guided, Radiation pneumonitis, Lung neoplasms

## Abstract

**Background:**

Computed tomography (CT) is the standard procedure for follow-up of non-small-cell lung cancer (NSCLC) after radiochemotherapy. CT has difficulties differentiating between tumor, atelectasis and radiation induced lung toxicity (RILT). Diffusion-weighted imaging (DWI) may enable a more accurate detection of vital tumor tissue. The aim of this study was to determine the diagnostic value of MRI versus CT in the follow-up of NSCLC.

**Methods:**

Twelve patients with NSCLC stages I-III scheduled for radiochemotherapy were enrolled in this prospective study. CT with i.v. contrast agent and non enhanced MRI were performed before and 3, 6 and 12 months after treatment. Standardized ROIs were used to determine the apparent diffusion weighted coefficient (ADC) within the tumor. Tumor size was assessed by the longest longitudinal diameter (LD) and tumor volume on DWI and CT. RILT was assessed on a 4-point-score in breath-triggered T2-TSE and CT.

**Results:**

There was no significant difference regarding LD and tumor volume between MRI and CT (*p* ≥ 0.6221, respectively *p* ≥ 0.25). Evaluation of RILT showed a very high correlation between MRI and CT at 3 (*r* = 0.8750) and 12 months (*r* = 0.903). Assessment of the ADC values suggested that patients with a good tumor response have higher ADC values than non-responders.

**Conclusions:**

DWI is equivalent to CT for tumor volume determination in patients with NSCLC during follow up. The extent of RILT can be reliably determined by MRI. DWI could become a beneficial method to assess tumor response more accurately. ADC values may be useful as a prognostic marker.

## Background

Stereotactic body radiotherapy (SBRT) has a high curative potential and offers excellent local tumor control in inoperable early stage NSCLC [1] while chemoradiotherapy is the standard treatment for inoperable, locally advanced NSCLC [2].

In stage III substantial improvements have been achieved during the past two decades with 5-year survival rates now surpassing 30% [3]. Yet, even with effective newer standards in radiotherapy (the integration of FDG-PET/CT based treatment planning, the application of involved-field target volume concepts to spare organs at risk and the use of intensity modulated radiotherapy [IMRT]) local tumor control still remains unsatisfactory. An actuarial locoregional recurrence rate of 40% after 3 years was reported by Garg et al. [4], in another retrospective report of Kandi et al. 67 of 137 patients (48,9%) experienced a locoregional recurrence after chemoradiotherapy [5]. An early diagnosis of recurrence or detecting a lack of tumor response is essential to provide the patient with an alternative treatment approach such as immunotherapy. For this reason, surveillance is important for monitoring the primary tumor site and detecting metastases.

Several oncological societies such as the National Comprehensive Cancer Network (NCCN) and the American College of Chest Physicians (ACCP) have generated guidelines for post-treatment surveillance imaging of NSCLC [6, 7]. The recommendations stated in these guidelines are based on low-grade evidence [8] and most existing studies address post-operative rather than post-radiation follow up.

The currently available guidelines recommend computed tomography (CT) of the chest as standard modality every 6–12 month after treatment of lung cancer for the first 2 years. CT is an established method that is easily available almost anywhere and will quickly generate high-resolution images with isotropic voxels that allow reconstructions in all spatial planes. However, follow up imaging by CT following radiation therapy can be challenging to interpret, because of radiation induced lung disease (RILT) or formation of tumor-atelectasis-complex [9]. In addition, iodine-containing contrast agent, which in turn carries risks, is obligatory in order to better evaluate mediastinal and hilar structures.

Thus, an examination which masters the differentiation of tumor atelectasis complex as well as distinguishing RILT versus recurrence would be advantageous. A suitable method that could fulfill this requirement is thoracic magnetic resonance tomography (MRI). In particular, diffusion weighted imaging (DWI) turns out to be promising. DWI is a technique which measures the movement of water molecules known as “brownian motion” using magnetic field gradients. Lesions with high cell density, like tumor tissue, appear bright in DWI imaging due to decreased diffusion. Recently published studies already pointed out the benefit of DWI in chest MRI for differential diagnosis of pulmonary nodules [10] as well as for initial staging of lung cancer and evaluation of lymph node status in particular [11, 12]. Furthermore, a study group could already show a good correspondence of initial tumor volume determination when comparing PET-CT and DWI [13]. However, there is only limited data about the value of DWI in the follow-up assessment of NSCLC after radiochemotherapy. In terms of tumor response, there is an ongoing debate about the predictive value of DWI in regard to apparent diffusion coefficient (ADC).

The aim of our study was to determine the diagnostic value of MRI, especially DWI, versus CT in the follow-up of NSCLC patients after radiochemotherapy. In addition to the assessment of the primary tumor course, a focus was placed on the evaluation of RILT.

## Methods

### Study population and inclusion criteria

Eligible patients were at least 18 years old and had a histological diagnosis of non-metastasized NSCLC (UICC stages I–III), had no contraindications to MRI, did not receive any previous antitumoral therapy and were allocated to receive–depending on tumor stage–either SBRT or definitive radiotherapy with concurrent chemotherapy. Written informed consent was obtained from all patients prior to study inclusion. This prospective study was conducted in accordance with the Helsinki declaration and was approved by the local ethics committee (blinded for review).

### Follow-up interval

The patients received both a planning CT and MRI before the start of radiotherapy. Follow-up CT and MRI were performed at 3, 6 and 12 months after radiotherapy. Follow-up CT examinations were performed in an outpatient setting according to the standard of care. All MRI examinations were performed at the Department of Diagnostic and Interventional Radiology. In order to ensure the best possible comparability of the two modalities, the time interval between the two examinations was kept as small as possible. The median interval between the acquisition of the CT and the MRI was 8 days (range 5–12 days) for the pretherapeutic examinations, 7 days (range 1–34) for the 3-month follow-up, 7 days (range 1–33) for the 6-month follow-up and 4 days (range 0–13) for the 12-month follow-up.

### Acquisition of CT and MRI images

Patients received their initial planning CT in the Department of Radiotherapy and Radiation Oncology. The planning CT was a Philips BigBoreTM 120 kV scanner (Philips Medical Systems, Amsterdam, The Netherlands). Patients received iodinated intravenous contrast medium adapted to body weight. The slice thickness was 3 mm, images were acquired during free shallow breathing. Only patients with stage I NSCLC designated for SBRT received an additional 4D-CT. Outpatient follow-up CTs were performed in inspiratory breath hold and after body weight-adapted administration of iodinated contrast medium.

All MRI examinations were performed using the same 1.5 T MRI scanner (Magnetom Aera, Siemens, Erlangen, Germany). A Half Fourier Acquisition Single Shot Turbo Spin Echo (HASTE) sequence (TE = 91 ms, TR = 1000 ms, Flip-angle = 125°, averages = 1, slice thickness 5 mm, FOV = 285 × 380 mm, matrix = 320 × 192) was acquired in transverse and coronal planes. To generate diffusion- weighted images a single-shot echo planar diffusion-weighted sequence with Stejskal-Tanner diffusion encoding scheme using an inversion recovery for fat saturation (TR = 15,400 ms, TE = 75 ms, TI = 180 ms, PAT factor of 2, 3-scan trace (averaged), averages = 4, slice thickness 5 mm, FOV = 309 × 380 mm, matrix = 208 × 128 (interpolated to 208 × 256), no gap) was acquired. The real voxel size of the sequence was 1.5 × 3 × 5 mm^3^. Two b-values at b = 0 and b = 800 s/mm^2^ were acquired. Fusion Images were composed of the HASTE and the DWI. ADC maps and additional high b-value images at b = 1400 s/mm^2^ were calculated automatically by the scanner software, based on linear signal decay. Both HASTE and DWI sequences were acquired with the patient breathing freely; these sequences were subsequently coregistered for image fusion.

To ensure adequate image quality, the DWI sequences were checked by the supervising medical assistant and the physician on duty immediately after their acquisition.

In the event of any imaging artifacts, the DWI-sequences were repeated (in altogether three examinations the DWI had to be repeated due to artifacts).

In addition, a respiratory gated T2-weighted sequence (TE = 106 ms, TR = 3692 ms, Flip- angle = 160°, averages = 2, 3 mm slice thickness, FOV = 277 × 370 mm, matrix = 384 × 202) was acquired in a transverse plane.

### Assessment of the tumor size, ADC values and RILT

Generally, all indicated tumor measurements relate to the primary tumor. The tumor size was evaluated according to RECIST 1.1. Thus, the longest longitudinal diameter (LD) of the lesion was measured and classified accordingly to its treatment response into “stable disease” (SD), “partial response” (PR), “progressive disease” (PD) and complete remission (CR). Depending on the course of the disease either baseline or nadir was used to compare and classify the tumor response. Furthermore, the extent of the primary cancer was determined by volumetric measurement. The tumor volume in its course was, in contrast to RECIST 1.1, always related to the baseline.

The examinations were evaluated by two observers with a work experience of 9 and 22 years in consensus. To generate the CT-derived tumor volume both soft tissue and lung window were regarded and the tumor was finally delineated in the lung window. For RECIST-evaluation, the longest longitudinal diameter of the primary cancer in lung window was measured. The delineation of the diffusion-weighted tumor volume was performed on diffusion weighted MR image sets and the measurement of the longest longitudinal diameter was also carried out on DWI. The performing observers were blinded for the corresponding CT tumor diameter and volume. The DWI-based contours were delineated visually and secondarily checked and–if necessary–adjusted for anatomical plausibility on the corresponding T2-weighted sequence.

DWI was also studied as a functional marker. The ADC maps were used as functional values. The tumor region with the lowest ADC was identified by consensus by the two radiologists and a standardized ROI of 100 mm^2^ was placed in this area. The ROIs had to be located completely within the tumor and were standardized to size for better comparability.

Consequently, the ROI had to be adjusted in some cases so that the average size of the ROI was 99.13 mm^2^ ± 6.91. T2-weighted MR images were used as a reference, to avoid the inclusion of necrotic areas. The mean and standard deviation as well as minimum and maximum values of the ROIs were recorded.

Each study was examined for morphological signs of RILT. A 5-point score established for CT examinations [14] was adapted to a 4-point score to assess RILT (0 = no radiation pneumonitis, 1 = reticular lung parenchyma changes, 2 = inhomogeneous consolidation, 3 = homogeneous consolidations). The CT examinations were evaluated in the lung window and the MRI examinations on respiratory-triggered T2-TSE images by the two observer pairs. The classification into the respective score was made by consensus. Furthermore, the interobserver variability of the longest tumor diameter and the RILT score was determined.

The evaluation of the longest longitudinal diameters, ADC values and RILT was done on a PACS workstation (SECTRA IDS 7 workstation, Sectra AB, Linkoeping, Sweden). Volume analysis was performed using a medical imaging software (Osirix MD 6.0, Pixmeo Sarl, Switzerland).

### Statistics

Statistical analysis was performed using GraphPad Prism (Prism® 8 for Mac, Version 8.00, GraphPad Software Inc., San Diego, CA, USA). Values are displayed as median and range (min. to max.) because normal distribution was not assumed. Nonparametric data was further analyzed with a Wilcoxon matched-pairs signed rank test. Correlation for non-normal distributed values was tested using the Spearman correlation test (R_S_). For comparison of the 4 ADC timepoints a mixed-effect analysis was performed with a Tukey test afterwards. Interobserver variability was evaluated using weighted kappa for ordinal variables. The assessment of agreement was made according to Landis et al. [15]. For continuous data, reliability was assessed using the intraclass correlation coefficient (ICC), which is commonly considered very good when greater than 0.90 [16]. The significance level was defined as *p* < 0.05.

## Results

### Patients’ and treatment characteristics

Twelve patients (3 female, 9 male) with histologically proven NSCLC were included in the study between July 2013 and November 2015. The median age was 68.5 years (range 42–79 years). One patient had two synchronous lesions of NSCLC (in both upper lobes), which were evaluated separately. TNM stages, histologies, UICC-stages, the localization of the primary tumor and follow-up classification according to RECIST 1.1 are shown in Table 1. The distribution of UICC-stages and histology was as follows: IA (*n* = 3), IIB (*n* = 1), IIIA (n = 3) and IIIB (n = 3) and IIIC (*n* = 2); squamous cell cancer (*n* = 8), adenocarcinoma (*n* = 4 whereof two were present in one patient) and ‘not otherwise specified’ (n = 1). In 3 patients with early stages (one of them with two lesions) stereotactic body radiotherapy was indicated. These patients received 4 × 12 Gy (equaling a biologically effective dose for an α/β - value of 10 [BED_10_] of 105.6 Gy), prescribed to the surrounding 80% isodose line of the PTV. Definitive radiochemotherapy was planned for all other patients with locally advanced stages using intensity modulated radiotherapy (IMRT) with a median isocenter dose of 60 Gy (range 58–66.6; single doses 1.8–2.0 Gy). For all patients (median PTV size of 484 mL [24.5–1407]), dosimetric parameters for organs at risk were as follows (given as median values): mean lung dose 14.9 Gy (4–20.8), V20 (total lung) 26% (3–30.2), V5 (total lung) 63.5% (17–88), mean esophageal dose 27.7 Gy (2.1–37.1), mean heart dose 6.85 Gy (0.1–33.5), spinal cord – maximum dose 37.3 Gy (1.8–42.8).

**Table 1:** Representation of the TNM stage, histology, the UICC stage, the localization of the primary tumor as well as the classification of the follow-up investigations according to RECIST 1.1. Note – PEC = squamous cell carcinoma, Adeno = Adeno carcinoma, SD = stable disease, PD = Progessive Disease, PR = Partial Remission, n.d. = not detectable, Pneumonitis = not detectable because of severe pneumonitis, m.a. = missed appointment

Case	TNM	Histology	Primarius	UICC	3 month CT	3 month DWI	6 months CT	6 months DWI	12 months CT	12 months CT	12 months DWI
1	T4 N2 M0	PEC	right upper lobe	III B	SD	SD	SD	m.a.	deceased	deceased	deceased
2	T3 N0 M0	PEC	middle lobe	II B	PR	SD	SD	CR	n.d. fibrosis	Pneumonitis	CR
3	T1b N0 M0	PEC	left lower lobe	I A	SD	PR	PD	PD	PD	PD	PD
4	T4 N3 M0	PEC	left centrally	III C	PD	PD	deceased	deceased	deceased	deceased	deceased
5	T4 N2 M0	PEC	right upper lobe	III B	PR	PR	SD	PD	n.d. fibrosis	Pneumonitis	m.a.
6	T4 N3 M0	Adeno	left upper lobe	III C	PR	PR	resection	resection	resection	resection	resection
7	T2a N2 M0	PEC	right lower lobe	III A	PR	PR	Pneumonitis	CR	n.d. fibrosis	Pneumonitis	CR
8	T1b N0 M0	-	left upper lobe	I A	PR	SD	SD	PR	deceased	deceased	deceased
9	T4 N2 M0	Adeno	right upper lobe	III B	PR	PR	Pneumonitis	PR	n.d. fibrosis	Pneumonitis	PR
10	T1b N0 M0	Adeno	right upper lobe	IA	SD	SD	SD	PR	m.a.	m.a.	m.a.
10	T1b N0 M0	Adeno	left upper lobe	I A	SD	SD	Pneumonitis	PR	m.a.	m.a.	m.a.
11	T1a N2 M0	PEC	right centrally	III A	PR	PR	PR	n.d.	SD	SD	PR
12	T2a N2 M0	PEC	left centrally	III A	PR	PR	SD	PR	SD	SD	PR

### Assessment of tumor size using RECIST 1.1/ LD

**Fig. 1:**
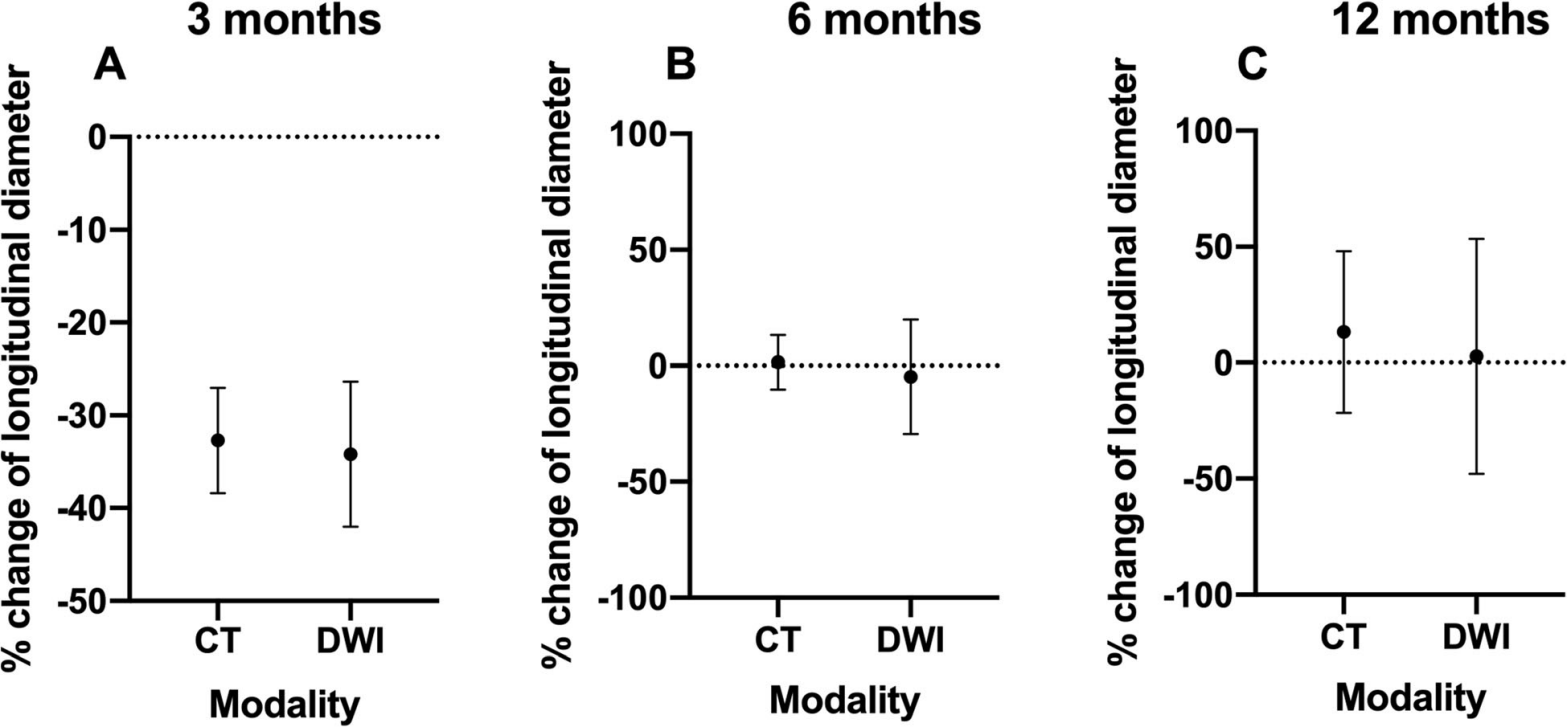
Representation of the percent change in the longitudinal diameter (LD) 3 (**a**), 6 (**b**) and 12 (**c**) months after initiation of therapy. Depending on the therapeutic response the reference value used to determine the percentage change in tumor response according to RECIST 1.1 are baseline or Nadir. All three panels show no statistically significant difference in the assessment of the LD. Values are displayed as mean and SEM

Figure 1 shows tumor response in CT and DWI over the three follow-up examinations.

#### 3-month follow-up

After 3 months, 13 CT and 12 DWI data sets were available. A total of 12 data pairs were comparable. In one patient the tumor could no longer be clearly identified by DWI; however, in this case, there was no difference in tumor response regarding RECIST 1.1 due to newly occurring distant metastasis. When assessing tumor response, there were 10 matches between CT and DWI as follows: PR (*n* = 6), SD (*n* = 3) and PD (*n* = 1). Two cases were discrepant, as CT classified them as PR and DWI as SD. However, in both cases of DWI only two percentage points were missing in order to classify them also as PR. A single reverse case in which DWI classified tumor response as PR and CT classified it as SD occurred.

The median percentage size changes of the tumor showed no significant difference between CT (− 33.33 [− 72.0–5.0]) and DWI (− 31.86 [− 76.0–13.92) (*p* = 0.6221).

#### 6 -month follow-up

After 6 months, 8 CT and 7 DWI data sets were available. A total of 5 data pairs were comparable. One patient had died of his tumor disease during this follow up interval.

Another patient left the study because of a resectable tumor after treatment initiation.

**Fig. 2:**
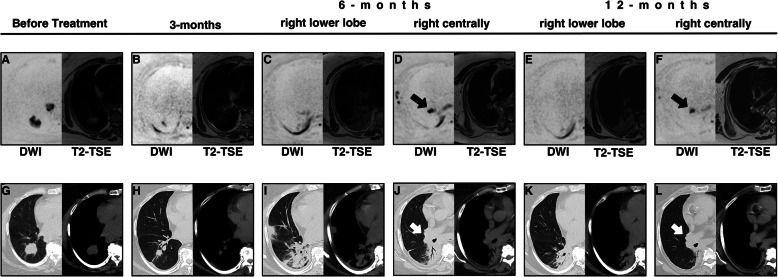
Presentation of the disease process before treatment and in the course of the three follow-ups. **a**-**f** shows the MRI sequences inverted DWI (*b*-value = 800 s/mm^2^) and T2-TSE in a transversal plane. G-L shows the corresponding CTs in lung and soft tissue window. The time points 6 and 12 months are divided into two anatomical regions (right lower lobe and right centrally). The tumor response after 3 months can be assessed in the DWI (**b**) as accurately as in the CT (**h**). After 6 months, severe radiation pneumonitis develops. Their extent can be determined both by MRI (**c**) and CT (**i**). Within pneumonitis it is difficult to make a statement on tumor response on CT (**i**). In the DWI (**c**), however, one can not detect any suspicious signal in the right lower lobe at this time. In addition, the DWI (**d**), in contrast to the CT (**j**) already delineates a suspicious signal on the right hilum. After 12 months, the findings after 6 months for pneumonitis in the right lower lobe and the suspicious lesion on the right hilum are reaffirmed. The extent of pneumonitis shows a good correlation between MRI (**e**) and CT (**k**) even after 12 months. The suspicious diffusion restriction in the right hilum (**f**) can still be clearly seen, whereas in CT (**l**) a delineation is much more difficult. After the study, the patient developed a right hilar recurrence

Another patient with SD on CT follow-up did not turn up for his MRI appointment. In another patient with SD after CT evaluation, the tumor could no longer be delineated on MRI (CR); retrospectively it could be shown that this patient was free from recurrence until the end of the study. Due to a pronounced fibrosis, no evaluation of the tumor in the right lower lobe was possible in one patient on CT. Interestingly, no considerable diffusion restriction could be seen in the corresponding DWI dataset in this area; however, at this time, in contrast to the CT scan, a diffusion-restricted lesion on the right hilar side was detectable; here the patient developed a recurrence (Fig. 2). In addition, DWI was able to classify 2 patients reliably as PR, whilst no tumor evaluation was possible on CT for these patients due to pneumonitis. One patient was scored on CT as a PR, at which time no tumor was assessable in the DWI. One patient had PD on both DWI and CT. Three cases were discrepant, as CT classified them as SD and DWI as PR, whereas the percentages of the two modalities were in part close to the threshold between PR and SD. The CT classified one patient as SD, which the DWI contrarily classified as PD; retrospectively, the patient developed a recurrence and died after the study.

The median percentage size changes of the tumor showed no significant difference between CT (4.23 [− 45.83–68.95]) and DWI (− 35.15 [− 48.46–122.8) (*p* > 0.99).

#### 12-month follow-up

After 12 months, 3 CT and 4 DWI data sets were available. A total of 3 data pairs were comparable. In the meantime, altogether three patients had died; two patients due to tumor progress and one patient due to exacerbated COPD. One patient did not show up at the MRI and the CT appointment, another one missed only the MRI appointment. Due to a pronounced fibrosis, no reliable evaluation of the tumor was possible in two patients on CT images, whereas DWI showed no suspect signal in these two patients (CR). The clinical course made it most likely that the diagnosis of CR was correct. In another case fibrosis did not allow for a clear statement regarding tumor size on CT images. The corresponding DWI showed a PR. Again, this diagnosis was confirmed by further follow up. Two patients were scored as SD on CT, whereas being rated as PR by DWI. However, the CT percentage values of those two patients were again close to the PR threshold. One patient had PD in both CT and DWI.

The median percentage size changes of the tumor showed no significant difference between CT (− 20.00 [− 23.00–82.75]) and DWI (− 39.53 [− 63.56–153.6) (*p* > 0.99).

### Assessment of tumor volume

**Fig. 3:**
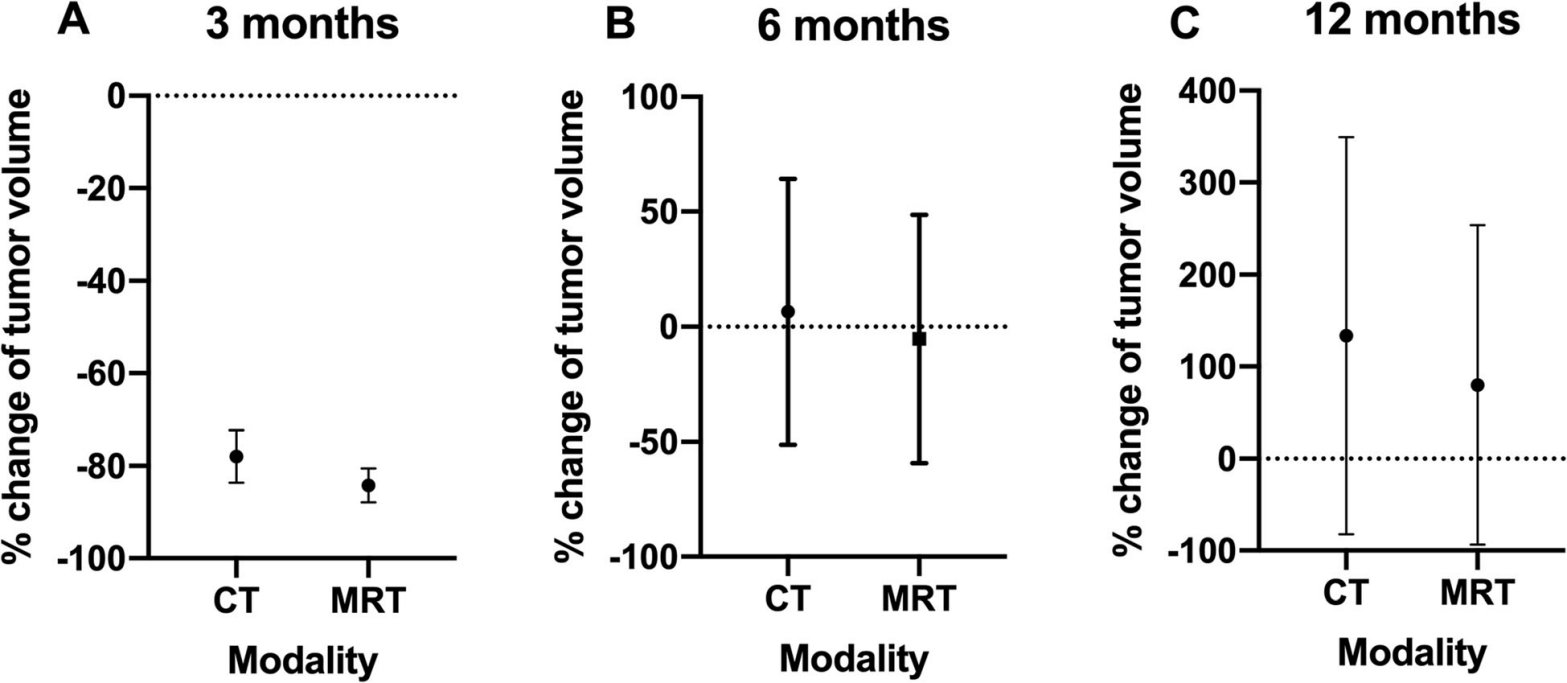
Representation of the percent change in tumor volume 3 (**a**), 6 (**b**) and 12 (**c**) months after initiation of therapy. The reference value used to determine the percentage change in tumor response is always the baseline. All three panels show no statistically significant difference in the assessment of the tumor volume. Values are displayed as mean and SEM

Figure 3 shows the development of tumor volume in CT and DWI over the course of time.

Available CT and DWI data sets as well as comparable data pairs are the same as in the assessment of tumor size using RECIST 1.1/LD.

At 3 (*p* = 0.5771), 6 (*p* = 0.3125) and 12 (*p* = 0.2500) months, there was no significant difference between CT and DWI in terms of tumor volume. At 3 months, the percentage change in tumor volume was − 84.16 [− 95.74 - -36.43) on CT and − 91.73 [− 95.84 - -59.69] on DWI. At 6 months, the percent change in tumor volume was − 29.87 [− 83.96–228.6) on CT and − 34.19 [− 88.79–204.5] on DWI. And at 12 months, the percent change in tumor volume was − 68.29 [− 95.88–564.7] on CT and – 89.11 [− 98.26–427.1] on DWI.

### Assessment of RILT

Table 2 shows by means of a contingency table the frequency distribution of the pneumonitis scores determined in CT and MRI (T2-TSE).

**Table 2:** Comparison of the absolute percentages of the CT and MRI pneumonitis score at 3, 6 and 12 months after the end of treatment. After 3 and 12 months there was a very good correlation. However, there was only a moderate correlation after 6 months. Overall, the score on MRI tends to be slightly lower than in the corresponding CT. Absolute frequencies are written in parenthesis behind the corresponding percentage value

Pneumonitis Score 3-months follow up					Pneumonitis Score 6-months follow up				Pneumonitis Score 12-months follow up			
Percentage of grand total	MRI 0	MRI 1	MRI 2	MRI 3	MRI 0	MRI 1	MRI 2	MRI 3	MRI 0	MRI 1	MRI 2	MRI 3
**CT 0**	35.71 (5)	7.14 (1)	0.00 (0)	0.00 (0)	10.00 (1)	0.00 (0)	0.00 (0)	0.00 (0)	33.33 (2)	0.00 (0)	0.00 (0)	0.00 (0)
**CT 1**	7.14 (1)	7.14 (1)	0.00 (0)	0.00 (0)	0.00 (0)	10.00 (1)	0.00 (0)	0.00 (0)	0.00 (0)	0.00 (0)	0.00 (0)	0.00 (0)
**CT 2**	0.00 (0)	0.00 (0)	14.29 (2)	0.00 (0)	0.00 (0)	10.00 (1)	20.00 (2)	0.00 (0)	0.00 (0)	0.00 (0)	16.67 (1)	0.00 (0)
**CT 3**	0.00 (0)	0.00 (0)	28.57 (4)	0.00 (0)	0.00 (0)	20.00 (2)	20.00 (2)	10.00 (1)	0.00 (0)	0.00 (0)	16.67 (1)	33.33 (2)

A total of 13 data pairs were comparable after 3 months. At the 6 months follow up 10 data pairs were comparable and at the 12 months follow up there were 6 data sets available.

The classification of pneumonitis on CT and MRI correlated very well after 3 months (r = 0.88). The median score was 1 [0–3] (mean with Std. deviation 1.15 ± 1.28) on CT and 1 on MRI [0–2] (mean with Std. deviation 0.92 ± 0.95). After 6 months, there was only a moderate correlation between the two modalities (r = 0.50). The median score was 3.0 [0–3] (mean with Std. deviation 2.27 ± 1.01) on CT and 1.5 on MRI [0–3] (mean with Std. deviation 1.5 ± 0.85). After 12 months, the correlation was again very good (r = 0.90). The median score was 2.5 [0–3] (mean with Std. deviation 1.83 ± 1.47) on CT and 2.0 on MRI [0–3] (mean with Std. deviation 1.6 ± 1.37).

### Functional imaging/ADC

**Fig. 4:**
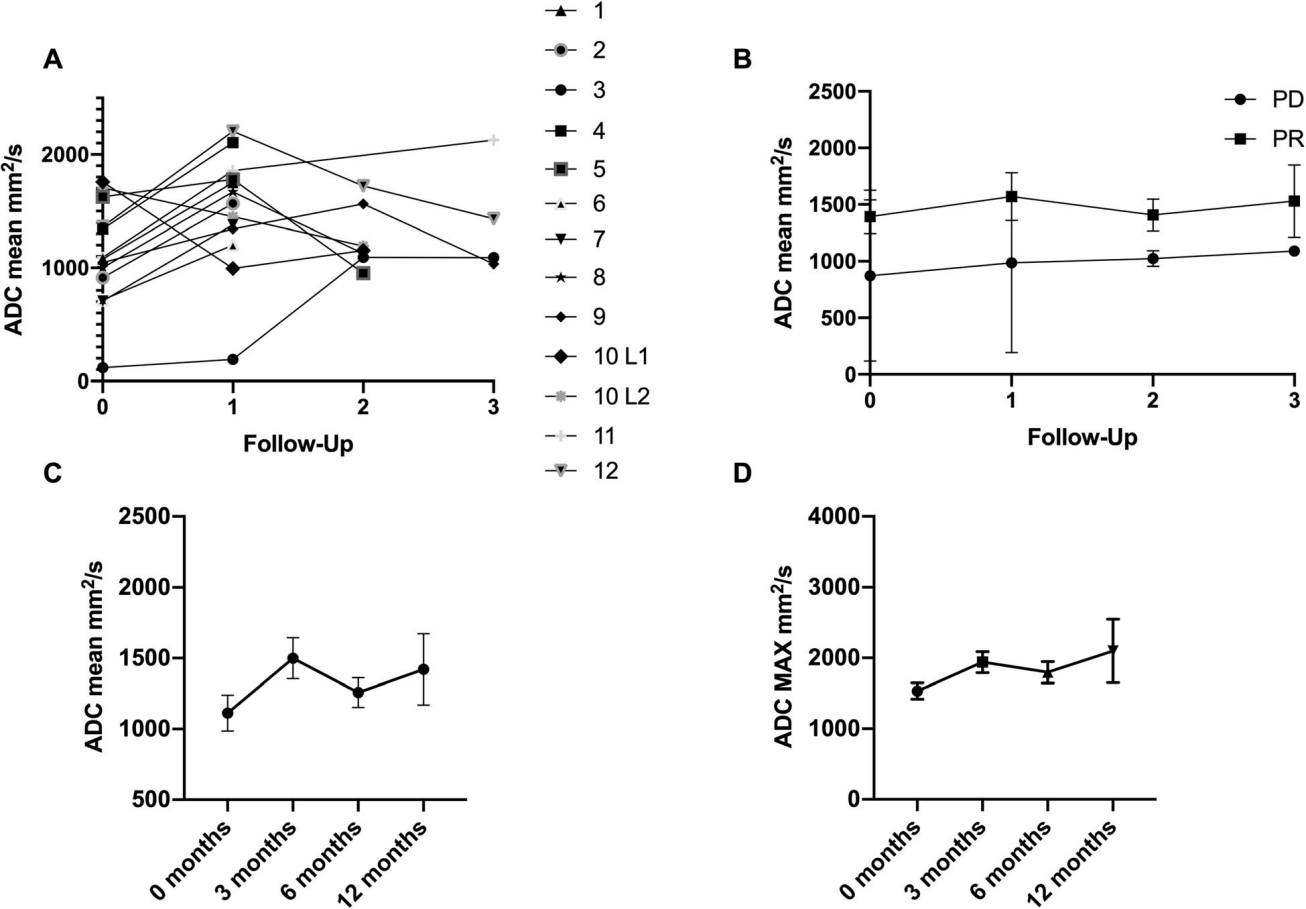
Panel **a** shows the course of the ADC values for all patients (cases 1–14; case 12 with two lesions) separately. Panel **b** divided the patients into a Progress (PD) and a Remission (PR) group. Patients in the progress group showed lower ADC values initially and throughout the course. In panel **c** and **d**, the mean and maximum (MAX) ADC values of all patients were averaged seperatly for each time point and presented in follow-up. In both Panel **c** and **d** there is a tendency for the ADC value to increase over time. Values in Panel **c** and **d** are displayed as mean and SEM

The mean ADC values of each patient in the course of the 4 timepoints are shown in Fig. 4 Panel a. Averaging the ADC values over the 4 time points revealed no statistically significant difference for either the ADC mean (*p* = 0.15) or the ADC maximum (*p* = 0.16) (Fig. 4 Panel c/d). However, an increasing trend could be observed for both values: ADC mean (0-months: 1113 ± 127.0, 3 months 1500 ± 144.6, 6 months 1258 ± 104.8 and 12 months 1421 ± 251.5) and ADC MAX (0-months: 1533 ± 133.9, 3 months 1940 ± 147.9, 6 months 1797 ± 150.9 and 12 months 2100 ± 477.8). Finally, the patient collective was divided into two groups: one with therapy response (PR) and another one with progressive disease (PD). However, it was not possible to include all patients, since not every patient could be clearly assigned to one of the two groups. The group with response to therapy had a higher ADC value at all times than the group with PD (Fig. 4 Panel b). However, the difference was not statistically significant (*p* = 0.13).

### Interobserver variability

ICC for continuous data, longest diameter, yielded an excellent agreement (ICC = 0.996; 95% CI, 0.990–0.998). Weighted kappa for ordinal data, RILT Score, resulted in a substantial agreement 0.780 (95% CI, 0.592–0.959).

## Discussion

MRI of the chest had long been technically challenging due to the movement and breathing artifacts of the thoracic organs as well as the susceptibility artifacts caused by the interfaces between different tissues and the overall low proton density of the lung [17].

The continuous technical development in MRI with fast imaging techniques, resulting in protocols with scan times between 15 and 30 min [18], has made chest MRI an interesting alternative to CT [19, 20]. Regarding the low evidence of appropriate follow-up examinations in NSCLC, especially after chemoradiation [8], chest MRI might be the future solution. Our study is one of the first longitudinal investigations comparing CT and MRI after chemoradiation of NSCLC regarding morphological and functional parameters.

Prior studies have mainly focused on pretreatment comparison of CT or PET/CT and MRI [13, 18]. Fleckenstein et al. already demonstrated a high level of concordance between the pretreatment tumor volumes of PET-CT and DWI for radiotherapy-planning [13].

FDG-PET/CT has a high diagnostic accuracy in the detection of local tumor recurrences [21] and is often used, when – based on the follow-up CT – a recurrence is suspected [22]. Nevertheless, FDG-uptake is also enhanced in lung regions which show severe RILT. Therefore, the diagnosis of local lung recurrences in areas of RILT might be impaired in such cases. Also, FDG-PET/CT is usually more expensive and less available than DWI and thus contraindicated as a routine measure in the follow-up due to economic and logistic reasons.

We observed no statistically significant difference regarding the percentage change of the longest longitudinal diameter between CT and DWI at 3, 6 and 12 months (Fig. 1). The spread of the measurements within the two modalities can be explained most easily on the different initial tumor sizes and its responses. An inverse tumor response between CT and DWI was not observed. This is also reflected by the classification of the tumor response according to RECIST 1.1. There was a high concordance between DWI and CT regarding therapy response after 3 months resulting in PR (*n* = 6), SD (*n* = 3) and PD (*n* = 1). In the three discrepant cases only a few percentage points were missing for the transition from PR to SD or SD to PR. However, at 6 and 12 months, there is a diminishing correspondence of CT and DWI in terms of tumor response. This observation may have multifactorial causes, such as RILT with limited sensitivity of CT scans, deceased patients and missed appointments. However, in the follow-up at 6 and 12 months in individual cases, the DWI detected recurrences earlier than CT or excluded them with greater certainty (Table 1 [Cases 2 and 7] and Fig. 2). These results are in line with the study published in May 2019 by Usuda et al. [23]. In their study DWI was more accurate than CT in determining a response of recurrent lesions of lung cancer to chemotherapy and/or radiotherapy. Consistent with our study, they concluded that DWI may be able to identify residual cancer, thereby improving specificity and sensitivity.

Conventional response criteria like RECIST 1.1 have some limitations. There is an ongoing debate how accurate a unidimensional measurement can represent the real tumor burden due to varying and often highly irregular tumor shapes. Meanwhile, several studies have demonstrated that volume measurement in lung tumors is more reproducible than size measurement [24, 25]. In addition, the study by Zhang et al. proves that DWI has a more precise delineation of lung cancer while exhibiting higher reproducibility [26]. In our study there was no significant difference between tumor volumes as determined by CT and DWI at any of the three follow-up dates. The tumor volumes in the DWI tended to be slightly smaller than in their CT counterparts. We identified the more precise demarcation of the tumor against atelectatic lung tissue and parenchymal changes in pneumonitis as the major cause for this discrepancy. There is a lack of data comparing CT and DWI tumor volumes in the course of therapy after chemoradiation. A comparable study by Weiss et al. determined significantly larger tumor volumes by CT as compared to DWI in patients after chemoradiation [27]. However, the results are only partially comparable to the data presented here, since the working group around Weiss et al. chose follow up assessments at 3 and 6 weeks, thus focusing on early changes.

In the future, we will be particularly challenged by the assessment of tumor process under immunotherapy. However, initial studies are already showing the advantages of MRI/DWI in the assessment of tumor response already [28, 29].

In addition to assessing tumor response, the applied imaging modality should reliably indicate RILT. RILT typically occurs as early as 4 to 12 weeks after treatment and may transform into radiation fibrosis (which may also occur independently) after 6 months or later [30]. Although clinically debilitating pneumonitis (grade ≥ 3) after radiotherapy develops in less than 10 % of patients [31], imaging in commonly used scores, such as the LENT-SOMA Score from the European Organization for Research and Treatment of Cancer (EORTC) [32], plays a role in diagnosis and therefore therapy. Regarding the evaluation of RILT, some groups use functional investigations in MRI with xenon gas with quite impressive results [33]. Meanwhile, the parenchymal structure of the lungs can be adequately assessed by MRI, as was shown by Sileo et al. in patients suffering from cystic fibrosis [34]. To our knowledge, we hereby present the first investigation to examine the correlation of RILT scores determined by CT and MRI, respectively respiratory gated T2-weighted sequence, over a period of 1 year. At 3 and 12 months a high correlation of the RILT scores was found. However, at the early stage of fibrosis development at 6 months, only a moderate correlation was shown between the two modalities. At this stage, the CT achieved a higher score than the MRI, which may be a hint for its earlier detection of reticular lung parenchyma changes. Overall, however, it can be concluded that respiratory gated T2-weighted sequence can adequately assess the ultrastructure of the lungs in the early and late phase after chemoradiation to diagnose or exclude RILT. Differentiating between treatment effects like RILT or tumor-atelectasis-complex and residual or recurrent tumor, is challenging [35]. Like aforementioned, in some cases of the presented group, by using DWI, as compared to CT, we were able to delineate recurrences earlier and to more reliably rule out recurrence within lung parenchyma altered by RILT (Fig. 2). These results are in line with a study of Munoz-Schuffenegger et al. in which they could prove that DWI confirmed the suspicion of local recurrence in patients with highly suspicious CT scans [36]. Furthermore, DWI/ADC not only provides these important additional informations but might also be a prognostic factor.

Looking at the individual mean ADC values of patients over the time course, no clear pattern could be observed in our study (Fig. 4a). However, averaging the ADC values of all patients at each time point mean and maximum ADC showed a tendency to increase (Fig. 4c/d). Our results are consistent with prior studies which demonstrated a significant ADC increase after chemoradiation and chemotherapy [27, 37, 38]. Weiss et al. showed that patients with survival < 12 months had a lower increase in ADC values compared to longer-lived patients [27]. Sampath et al. could demonstrate that an ADC increase of 40% at 1 month after SBRT for NSCLC is associated with a higher rate of local failure [39]. In contrast, non-responders in the study by Chang et al. had a slight decrease in ADC, whereas responders had a relatively steeper increase of ADC [37]. As opposed to the latter data, after formation of a PD and PR group, we were unable to detect a significant increase or decrease in the mean ADC between the two groups (Fig. 4b), which could be due to the small sample size. However, both the pretherapeutic and the mean ADC values over the course tend to be lower in non-responders (PD group). In agreement with our findings, Shintani et al. and Iizuka et al. found that low ADC on pre-treatment MRI were associated with local recurrence and poor disease progression [40, 41]. Yet, Ohno et al. reported contradictory findings in patients in whom higher ADC on pretreatment MRI were significantly associated with poor prognosis [42].

The discrepancies in the predictive power of the ADC may in part be due to the non-uniform measurement. Depending on the study, the mean, minimum or maximum ADC value is used. Furthermore, until now there is no clear definition of where within the tumor one should place the ADC ROI. Further studies are needed to establish a uniform and reproduceable measurement of the ADC and thus to substantiate its prognostic value.

Beside of all of these capabilities MRI offers in imaging of the NSCLC, the acquisition time of this modality has to be viewed critically especially in comparison with CT. As mentioned in the first section of the Discussion, the MRI protocol takes about 15–30 min (median duration 33 min, range [max to min] 19 min to 1 h and 13 min). Compared to a CT scan of the thorax with an acquisition time of only a few seconds for the actual scan and a few minutes for the entire examination, this is of course a considerable effort, especially for patients with impaired lung function. However, the MRI protocol can certainly be optimized by removing, respectively limiting the time-consuming breath-triggered T2-TSE to the target areas, because acquisition of the whole thorax can take between 15 and 30 min depending on the patient’s body height and breathing variability. If assessment of the ultra-structure of the lung is not required, DWI/ADC in combination with T2-HASTE, both only taking about 5 min for image acquisition, could be a solution for thorax imaging regarding T and N stadium.

Our study had some limitations. First, it is a single center study with a small number of patients. Additionally, some patients did not complete scanning schedule and we can’t exclude the possibility that this might have skewed the results.

## Conclusion

In conclusion we present an initial longitudinal study, which demonstrates that after chemoradiation therapy response determined by RECIST 1.1 and tumor volume measurement can be done by DWI yielding similar results to CT. In addition, the presented study is one of the first to describe typical changes of RILT in the early and late phase as diagnosed with MRI as compared to the gold standard of CT. Thus, MRI including DWI, bears a strong potential for improved detection of an inadequate response to radiotherapy or early recurrences. In regard to the potential prognostic value of ADC measurements further investigations are necessary.

## Data Availability

The datasets used and analysed during the current study are available from the corresponding author on reasonable request.

## Abbreviations

ACCP: American College of Chest Physicians
ADC: Apparent diffusion coefficient
CR: Complete remission
CT: Computed tomography
DWI: Diffusion weighted imaging
EORTC: European Organization for Research and Treatment of Cancer
LD: Longitudinal diameter
MRI: Magnetic resonance imaging
NCCN: National Comprehensive Cancer Network
NSCLC: Non small cell lung cancer
PD: Progressive disease
PR: Partial remission
RILT: Radiation induced lung toxicity
SBRT: Stereotactic body radiotherapy
SD: Stable disease
UICC: Union for International Cancer Control
