# Correction to: Diffusion-weighted MRI improves response assessment after definitive radiotherapy in patients with NSCLC

**DOI:** 10.1186/s40644-021-00386-7

**Published:** 2021-02-09

**Authors:** Philippe Jagoda, Jochen Fleckenstein, Mathias Sonnhoff, Günther Schneider, Christian Ruebe, Arno Buecker, Jonas Stroeder

**Affiliations:** 1 Clinic for Diagnostic and Interventional Radiology, Saarland University Medical Center, Kirrberger Str. 1, 66421 Homburg, Saar Germany; 2 Department of Radiotherapy and Radiation Oncology, Saarland University Medical Center, Kirrberger Str. Geb. 6.5, 66421 Homburg, Saar Germany

**Correction to: Cancer Imaging 21, 15 (2021)**

**https://doi.org/10.1186/s40644-021-00384-9**

After publication of the original article [1], the authors identified that authors’ names have been wrongly marked. The given name and family name were erroneously transposed.

The incorrect authors’ names are:

<GivenName> Jagoda </GivenName> < FamilyName> Philippe </FamilyName>

< GivenName> Fleckenstein </GivenName> < FamilyName> Jochen </FamilyName>

<GivenName> Sonnhoff </GivenName> < FamilyName> Mathias </FamilyName>

<GivenName> Schneider </GivenName> < FamilyName> Günther </FamilyName>

<GivenName> Ruebe </GivenName> < FamilyName> Christian </FamilyName>

<GivenName> Buecker </GivenName> < FamilyName> Arno </FamilyName>

<GivenName> Stroeder </GivenName> < FamilyName> Jonas </FamilyName>

The correct authors’ names are:

<GivenName> Philippe </GivenName> < FamilyName> Jagoda </FamilyName>

<GivenName> Jochen </GivenName> < FamilyName> Fleckenstein </FamilyName>

<GivenName> Mathias </GivenName> < FamilyName> Sonnhoff </FamilyName>

<GivenName> Günther </GivenName> < FamilyName> Schneider </FamilyName>

<GivenName> Christian </GivenName> < FamilyName>Ruebe </FamilyName>

<GivenName> Arno </GivenName> < FamilyName>Bueker</FamilyName>

<GivenName> Jonas </GivenName> < FamilyName>Stroeder</FamilyName>

The original article has been corrected.
